# Genome-wide association analyses using multilocus models on bananas (*Musa* spp.) reveal candidate genes related to morphology, fruit quality, and yield

**DOI:** 10.1093/g3journal/jkae108

**Published:** 2024-05-22

**Authors:** Jaime Andrés Osorio-Guarin, Janet Higgins, Deisy Lisseth Toloza-Moreno, Federica Di Palma, Ayda Lilia Enriquez Valencia, Fernando Riveros Munévar, José J De Vega, Roxana Yockteng

**Affiliations:** Centro de Investigación Tibaitatá, Corporación Colombiana de Investigación Agropecuaria, AGROSAVIA, Km 14 vía Mosquera, Cundinamarca 250047, Colombia; Earlham Institute, Norwich Research Park, NR4 7UZ Norwich, UK; Centro de Investigación Tibaitatá, Corporación Colombiana de Investigación Agropecuaria, AGROSAVIA, Km 14 vía Mosquera, Cundinamarca 250047, Colombia; Earlham Institute, Norwich Research Park, NR4 7UZ Norwich, UK; Centro de Investigación Palmira, Corporación Colombiana de Investigación Agropecuaria, AGROSAVIA, Palmira, Valle del Cauca 763533, Colombia; Facultad de Psicología y Ciencias del Comportamiento, Universidad de La Sabana, Chía, Cundinamarca 250001, Colombia; Earlham Institute, Norwich Research Park, NR4 7UZ Norwich, UK; Centro de Investigación Tibaitatá, Corporación Colombiana de Investigación Agropecuaria, AGROSAVIA, Km 14 vía Mosquera, Cundinamarca 250047, Colombia; Institut de Systématique, Evolution, Biodiversité-UMR-CNRS 7205, Muséum National d´Histoire Naturelle, Paris, Ile 75005, France

**Keywords:** association mapping, germplasm, *Musa;* skim sequencing

## Abstract

Bananas (*Musa* spp.) are an essential fruit worldwide and rank as the fourth most significant food crop for addressing malnutrition due to their rich nutrients and starch content. The potential of their genetic diversity remains untapped due to limited molecular breeding tools. Our study examined a phenotypically diverse group of 124 accessions from the Colombian Musaceae Collection conserved in AGROSAVIA. We assessed 12 traits categorized into morphology, fruit quality, and yield, alongside sequence data. Our sequencing efforts provided valuable insights, with an average depth of about 7× per accession, resulting in 187,133 single-nucleotide polymorphisms (SNPs) against *Musa acuminata* (A genome) and 220,451 against *Musa balbisiana* (B genome). Population structure analysis grouped samples into four and five clusters based on the reference genome. By using different association models, we identified marker–trait associations (MTAs). The mixed linear model revealed four MTAs, while the Bayesian-information and linkage-disequilibrium iteratively nested keyway and fixed and random model for circulating probability unification models identified 82 and 70 MTAs, respectively. We identified 38 and 40 candidate genes in linkage proximity to significant MTAs for the A genome and B genome, respectively. Our findings provide insights into the genetic underpinnings of morphology, fruit quality, and yield. Once validated, the SNP markers and candidate genes can potentially drive advancements in genomic-guided breeding strategies to enhance banana crop improvement.

## Introduction

Tropical species of the *Musa* genus produce starchy fruit, consumed worldwide, and is of great importance for food security. In 2022, the total production of bananas globally reached 140 million tons (FAO 2022). In addition, bananas are Colombia's third most important agricultural export, behind coffee and ornamental flowers, accounting for ∼2.1 million tons in 2022 (FAO 2022). The two principal *Musa* fruits are bananas and plantains. Most edible *Musa* cultivars are hybrid, polyploid, and vegetatively propagated because they usually have parthenocarpic fruits. This feature causes low fertility and hinders the genetic improvement of the crop (Sardos *et al.* 2016).

*Musa*, domesticated in Southeast Asia, was introduced into Africa, the Americas, and other parts of the world (de Jesus *et al.* 2013). Many *Musa* varieties produce fruits with high caloric content. Plant parts are used as food, fodder, fiber, and traditional medicines (Panda *et al.* 2020). Cultivated bananas arose from a complex domestication scheme involving several taxa, including different subspecies of *Musa acuminata* Colla (A genome) and *Musa balbisiana* Colla (B genome) (Christelová *et al.* 2017). Modern cultivars contain combinations with various levels of ploidy produced through unbalanced meiosis, such as diploid (AA; BB; or AB; 2*n* = 2× = 22), triploid (AAA; AAB; or ABB; 2*n* = 3× = 33), and tetraploid (AAAA; AAAB; AABB; or ABBB; 2*n* = 4× = 44) (de Jesus *et al.* 2013).

Developing high-yielding varieties is essential to meet the food demand of a growing population. Precise improvement of complex quantitative traits needs the identification of associated genomic regions, like quantitative trait loci (QTLs), to enrich the gene diversity (Swarup *et al.* 2021). Genome-wide association study (GWAS) effectively identifies genes and QTLs based on the linkage disequilibrium (LD). This method has been widely used in several crops using genome-wide dense markers to predict candidate genes (Tibbs Cortes *et al.* 2021). The principal advantages of this method are as follows: (1) it uses diverse germplasm, making the procedure more efficient and less expensive than bi-parental QTL mapping, and (2) the high resolution and power of association studies (Tibbs Cortes *et al.* 2021).

GWAS can use single-nucleotide polymorphisms (SNPs) markers produced by high-throughput sequencing from reduced genome representation libraries [for example, genotyping by sequencing (GBS) and restriction site-associated DNA sequencing]. However, the decrease in sequencing costs has allowed the implementation of techniques such as skim sequencing (skim-seq), in which the whole genome of the studied species is sequenced at low coverage (Kumar *et al.* 2021). This technique is an effective tool for genotyping and identifying many SNPs associated with traits of interest for applied breeding programs (Adhikari *et al.* 2022).

In GWAS, differentiating genuine associations from false-positive marker–trait associations (MTAs) caused by population structure and kinship is challenging (Kaler and Purcell 2019). Single-locus GWAS (SL-GWAS) methods such as mixed linear model (MLM), which accounts for the random effect associated with kinship and covariates such as population structure but not for the association between markers, have been widely used to control spurious MTAs (Zhang *et al.* 2005). However, SL-GWAS models testing one locus at a time fail to model complex traits controlled simultaneously by numerous loci. Multiple critical-value test corrections are usually required to reduce false-positive rates for SL-GWAS (Zhang *et al.* 2019b). Multilocus GWAS (ML-GWAS) models can be used to improve the accuracy of results and overcome the SL-GWAS limitations, avoiding the confounding effects of population structure by accounting for kinship and principal components. Models like fixed and random model for circulating probability unification (FarmCPU) (Liu *et al.* 2016) provided higher statistical power and eliminated false-positive MTAs without compromising genuine associations. This model can include multiple markers simultaneously as covariates, partially removing the confounding effect of markers (which refers to a variable whose omission from a GWAS regression model will cause a spurious association between the genotype and the phenotype) and kinship. It employs the fixed-effect model (FEM) and random-effect model iteratively to remove confounding altogether. Besides, Bayesian-information and linkage-disequilibrium iteratively nested keyway (BLINK) (Huang *et al.* 2019) have been developed, which has a higher statistical power and is more time-efficient, reducing computing time by replacing random effect with a FEM.

During recent years, some GWASs have been conducted in *Musa* samples. Sardos *et al.* (2016), using a panel of 105 accessions and 5,544 SNP markers, reported 13 candidate genomic regions related to seedless phenotype using the MLM. Also, Nyine *et al*. (2019) conducted the first GWAS for bunch-weight components. In this study, the authors used 307 genotypes combined with 27,178 SNPs. Finally, they reported 25 genomic loci principally localized on chromosome 3 using the MLM.

In this work, we undertook a GWAS on morphology, fruit quality, and yield traits in 124 accessions from the Colombian Musaceae Collection (CMC) to (1) study the phenotypic variance and (2) detect genetic loci underlying the studied agronomic traits. The results should help to understand the genetic basis of morphology, fruit quality, and yield traits in promissory *Musa* cultivars to facilitate further genetic improvement through marker-assisted selection.

## Materials and methods

### Plant material

The plant material used is established in situ in the CMC. It contains *Musa* landraces and cultivars, administered by the Corporación Colombiana de Investigación Agropecuaria—AGROSAVIA at the Palmira Research Center, Valle del Cauca, Colombia (76°18′51.8″ W, 3°30′42.4″N). This center is located at 1,000 m.a.s.l. and presents an annual average temperature of 23°C, precipitation of 1,100 mm, and relative humidity of 75%. The 190 accessions of the collection (Higgins *et al.* 2023) were established in an area of 1.5 ha, in clay loam soils and flat topography, in five consecutive blocks separated by three meters. Each block comprised a productive site (mother plant, daughter, and granddaughter) of each accession (190 sites per block), meaning that there were five repetitions or productive sites per accession, one in each block. The organization within the block was in consecutive order of the field code for each accession, in the same way in all blocks. The agronomic management was based on cultural practices such as defoliation, elimination of new individuals, elimination of dry socks from the plant stem, and elimination of the pseudostem once the plant was harvested. The fertilization was based mainly on K and N supplementing, with supplemental irrigation carried out at field capacity, and weed was mechanically managed with a manual scythe. Phytosanitary management focuses on controlling the weevil complex with pseudostem traps and using entomopathogenic fungi.

Of this collection, we selected the 124-panel used in this study based on the availability of phenotypic data with a maximum of 20% missing data and the representation of different genetic groups. The association panel represents different genome ploidies (31 diploids, 79 triploids, and 14 tetraploids samples), which are grouped into 21 genetic subgroups and 13 genomic clonal varietal groups previously reported by Higgins *et al*. (2023) (Supplementary Table 1).

### DNA isolation, library preparation, and sequencing

Previously, the DNA of the 124 samples was extracted using the DNeasy Plant Mini Kit (QIAGEN, Germany), following the manufacturer's instructions. The concentration of the DNA was measured using a NanoDrop 1000 UV spectrophotometer (Thermo Scientific, Wilmington, USA), and the quality was verified by electrophoresis in 1% agarose.

Library preparation and genotypification were performed at Earlham Institute (Norwich, UK) using the skim-seq method. Libraries were sequenced on the *Illumina* NovaSeq sequencer using a standard *Illumina* library with 150-bp pair-end reads, as detailed in Higgins *et al*. (2023).

### Phenotypic characterization

The evaluated variables were grouped into morphology, fruit quality, and yield categories (Table 1, Supplementary Table 2). According to the commercial harvest criteria, all variables were recorded when the bunches reached physiological harvest maturity (Maguiña and Benigna 2019). Maturity is achieved when the fruits of the first hand of the bunch have a yellow hue, the fingers have lost their angularity or edges due to their filling, and the tips of the fingers have turned black. The variables were recorded in the second crop cycle, and the repetitions were from the different blocks planted in the field.

**Table 1.** Phenotypic traits of the *Musa* accessions used for analysis.

Category	Trait	Acronym	Units
**Morphology**	Pseudostem height	HT	Meters (m)
	Single leaf width	LBW	Centimeters (cm)
	Time from flowering to harvest	FTH	Days
**Fruit quality**	pH	pH	-
	Total soluble solids	TSS	°Brix
	Titratable acidity	TA	of g—organics acid/100 g of pulp
**Yield**	Hands weight	HW	Grams (g)
	Number of hands in a bunch	BH	Number
	Fruit length	FL	Centimeters (cm)
	Pulp percentage	PP	Percentage (%)
	Pulp dry weight percentage	PDW	
	Peel thickness	PT	Millimeters (mm)

Morphological variables such as pseudostem height (HT), single leaf blade width (LBW), and time from flowering to harvest (FTH) were recorded directly in the field. HT was measured in meters (m) from the stem base to the point of peduncle emergence, according to the descriptors of the International Plant Genetic Resources Institute (IPGRI-INIBAP/CIRAD 1996) and the *Musa*Net (Taxonomy Advisory Group 2010). Similarly, the LBW was recorded in centimeters (cm) with a tape measure at the maximum point. We calculated FTH in days as the time elapsed from flowering (when the last bracts fall from the most distal hand to display the female flowers) to the harvest of the bunch. The flowering date was marked at the emergence of the acorn using plastic tape hung from the peduncle, and each week was indicated by different tape colors. The harvest was carried out once the commercial harvest criteria were met (Maguiña and Benigna 2019).

The fruit quality variables, such as the content of total soluble solids (TSS), the acidity or alkalinity of a sample (pH), and the titratable acidity (TA), were recorded in the green stage to the bunch harvest by triplicate in the AGROSAVIA laboratory at the Research Center Palmira. For the preparation of the sample, 30 g of pulp from the central finger of the second hand of the bunch was liquefied for 2 min in 90 ml of distilled water and filtered using a membrane grade 40 with a pore size of 8 μm. To quantify TSS, we placed a sample drop in a digital refractometer (PAL-1 BRIX 0.0-53%). The TSS content, calculated in Brix degrees (°Brix), is composed of sugars (the most abundant), salts, acids, vitamin C, amino acids, and some pectins and interpreted as the percentage of sugar in the sample (Yanes 2018). The pH was determined using a Metrohm potentiometer (model 744 pH meter), and the TA was quantified by titration with an acid–base reaction, and the results were expressed in grams (g)—organics acid/100 g of pulp (Table 1).

The cluster was separated into stalk, rachis, acorn, hands, and fingers to determine the yield variables (Table 1). The quantification of the data was carried out in triplicate for each accession. The hand's weight (HW) was recorded in grams (g) on a digital scale (Mettler, g ± 0.01). The number of hands on a bunch (BH) was counted, and using the second hand emitted from each bunch, the number of fruits was determined. The fruit or finger length (FL) was measured in cm, selecting three fingers of the second hand of the bunch. The outer arc was measured with a tape measure (precision ± 1 mm) to the apex of the fruit without considering the pedicel. To determine the percentage of pulp dry matter weight (PDW) and the pulp percentage (PP), we used the second hand of the bunch. The PDW was calculated as the difference between the fresh and the dry pulp weight, dehydrated at 105°C for 48 h. The PP was calculated as the difference between the unpeeled and peeled fruit weights. We cut the fruit in half to determine peel thickness (PT) and separated the peel from the pulp. We measured in millimeters (mm) the PT in triplicate with a caliper or Pie King 6′ (0–150 mm) Mitutoyo 530-104 analog.

### Statistical analysis of phenotypic information

We calculated descriptive statistics of the phenotypic data of 12 traits for the 124 *Musa* accessions. An analysis of variance (ANOVA), with a significance level of *P* < 0.05, was performed to establish statistically significant differences between accessions according to their genome (AA, AAA, AAAA, AAAB, AAB, AABB, AB, and ABB). Tukey's multiple comparison test was used to establish statistically significant differences between pairs of means. Broad-sense heritability (h^2^_bs_) of all traits was calculated using the formula described by Allard (1960) as follows: h^2^_bs_ = [(σ^2^_G_)/(σ^2^_P_)] × 100, where σ^2^_G_ is the genotypic variance and σ^2^_P_ is the phenotypic variance. The correlation between the evaluated traits was calculated using Pearson's correlation coefficient (*r*; *P* ≤ 0.05) and principal component analysis (PCA) to assess the variable contribution in accounting for the variability in each principal component, and the relationship and the grouping between accessions were performed. Finally, a multiple linear regression analysis (*r^2^*) was performed to establish the predictor variables for three of the most important variables (one for each category): HT for morphology, TSS for fruit quality, and HW for yield. We used JASP v0.16.4 software (JASP Team 2023) for statistical analysis. The Factoextra package of R (Kassambara and Mundt 2020) was used to plot the PCA, the optical clusters, and the hierarchical dendrogram using the clustering method Silhouette.

### SNP calling

SNP discovery was previously performed by Higgins *et al*. (2023) with the GATK HaplotypeCaller v3.7.0 (McKenna *et al.* 2010) software using the alignments against *M. acuminata* doubled-haploid cv. Pahang accession version 4 (A genome) (Liu *et al.* 2023) and an *M. balbisiana* cv. Pisang Klutuk Wulung accession version 1.1 (B genome) (Wang *et al.* 2019).

### Population structure and LD analysis

We carried out the following analyses using the two genome references. The population structure was inferred using the maximum likelihood method on the Admixture v1.3 software (Alexander and Lange 2011) using values of *K* that varied from 1 to 10. The best *K* was selected using the lowest cross-validation error values. The accessions with a proportion of ancestry ≥ 0.5 were assigned to a unique cluster, while samples < 0.5 were assigned to a mixed cluster. The relative kinship coefficients of individual genotypes based on identity were estimated using the Loiselle method (Loiselle *et al.* 1995).

LD was calculated among all the possible pairs of SNPs and estimated using *r^2^* (squared allele frequency correlation). The LD block size of the whole genome was calculated by fixing the *r^2^* threshold at half LD decay using PopLDdecay software (Zhang *et al.* 2019a). Values *r^2^* were plotted as a function of genetic distance in kilobases (kb) using loess regression to visualize the LD decline in R.

### Genome-wide association study

With the adjusted means of the 12 phenotypic traits for each accession, we used different statistical GWAS models to identify candidate genes that may be associated with agronomic characteristics. The SNPs were subjected to a quality filter in VCFTools software (Danecek *et al.* 2011): data above 20% missing data and minor allele frequency (MAF) *<* 0.05% were discarded. We used GAPIT v3.0 package for R (Wang and Zhang 2021) to test the MLM approach for the SL-GWAS. We used FarmCPU and BLINK for the ML-GWAS. The admixture matrix was used to correct the population structure. The kinship matrix also accounted for the relationships among individuals.

The MLM equation was *y* = *si* + *Q* + *K* + *e*, where *y* corresponds to the phenotypic observations. The fixed effects that help to reduce false positives are the *Q*, considered as population structure, and the *K*, that is, the relationship between individuals, which is included as the kinship matrix. Finally, the *e* is the random vector of residual effects. The FarmCPU is an ML-GWAS analysis that performed fixed and random effects using the formula *y* = *si* + *S* + *e*. This model removes the confounding data using multiple markers as covariates. The obtained *K* value was used to select the associated markers with the maximum likelihood method. Finally, the BLINK model eliminates the computational complexity (Wang and Zhang 2021) using the Bayesian information content in a FEM.

The *P*-value obtained for each SNP was transformed into a base-10 logarithmic scale and later presented in circular Manhattan and quantile–quantile (QQ) plots. The significant associations between SNPs were corrected using the false discovery rate (FDR) correction. Only MTAs that exceeded the threshold value were reported in this study. We considered an MTA an SNP associated with a gene protein or transcription factor. The marker effect and phenotype variance (PV) explained (%) were also estimated for each MTA.

### SNP annotation

The candidate genes were searched within a 50-kb flanking region (approximately 50-kb upstream and 50-kb downstream) of the detected significant SNP using *the M. acuminata* DH Pahang (v4) and *M. balbisiana* DH-PKW (v1.1) from the JBrowse of the banana genome hub (https://banana-genome-hub.southgreen.fr/musa_acuminata_pahang_v4 and https://banana-genome-hub.southgreen.fr/musa_balbisiana_v1.1). We recognized the biological functions of genes/transcripts close to the significant SNPs by blasting the flanking sequences of candidate SNPs against the database of the National Center for Biotechnology Information (NCBI) (http://www.ncbi.nlm.nih.gov/). We also looked for potential loss-of-function alleles among the list of candidate genes by examining the predicted impact of the complete set of nucleotide variants located within genic regions using the software SnpEff v5.0 (Cingolani *et al.* 2012). The SNP-predicted effects were categorized by their impact as high (disruptive impact on the protein), moderate (nonsynonymous substitution), low (synonymous substitution), and modifier (with effects on noncoding regions).

## Results

### Phenotypic characterization of *Musa* accessions

The statistical descriptors for the morphological variables, such as HT and LBW, were similar, with values ranging from 2.27 m in accessions AAA to 2.98 m in the accession AABB and from 64.50 cm in accessions ABB to 82.90 cm for accessions AAAA (Supplementary Table 3). In addition, the traits related to fruit quality were similar between genomic groups. The pH ranged from 5.30 (AAAA) to 5.65 (AAB), and values of TSS were between 3.30 (AAAA) and 4.29 (AA) °Brix, except for AAAB, which had 5.13 (± 1.61) °Brix. TA values fell between 0.06 (ABB) and 0.09 (AAAB) g—organics acid/100 g of pulp.

The FTH trait ranged from 92.06 days on average for AA to 148.50 days for AABB. We also observed that fruit length (FL) and PT were higher in triploid and tetraploid accessions than in diploid accessions. The diploid AA had a mean value of 9.53 (± 1.12) cm in FL and 2.95 (± 0.63) mm in ST, and AB had 10.25 cm in FL and 2.60 mm in ST. Triploids AAA, AAB, and ABB had mean values for FL of 14.87 (± 2.67),17.07 (± 3.44), and 13.55 (± 1.79) cm, respectively, and for ST, AAA had means values of 3.88 (± 0.87), AAB 3.97 (± 1.17), and ABB 3.98 (± 0.60) mm (Supplementary Table 3).

As a result of the ANOVA, the Tukey test showed that some phenotypic traits presented significant differences according to the genome or ploidy level (Table 2). The attributes of the diploid genome AA were the most significantly different from those of the other genomic groups. Characteristics such as FTH, HW, and FL were lower in AA samples (92.06 ± 11.26 days, 7121.16 ± 2956.16 g, and 9.53 ± 1.12 cm, respectively), while PP was higher (66.78% ± 6.12, Supplementary Table 3). AAA triploid accessions and tetraploid (AAAA and AAAB) genomes were statistically different in traits such as FTH and PDW compared to AAB triploid accessions. However, quality traits (pH, TSS, and TA) did not present significant differences according to the genome of the accessions. Meanwhile, the traits’ h^2^_bs_ ranged from 25.1% for TA to 99.9% for PDW (Supplementary Table 3).

**Table 2.** Post hoc Tukey’s test based on ANOVA for phenotypic traits according to the genome of the *Musa* accessions.

Genome		Morphology trait			Quality trait			Yield trait					
		HT	LBW	FTH	pH	TSS	TA	HW	BH	FL	PP	PDW	ST
**AA**	**AAA**	0.631	0.088	**<0.001** *^c^*	1.000	0.892	1.000	**<0.001** *^c^*	0.203	**<0.001** *^c^*	**<0.001** *^c^*	**<0.001** *^c^*	**0**.**002***^b^*
	**AAAA**	0.999	**0**.**024***^a^*	**0**.**002***^b^*	1.000	0.885	1.000	**<0.001** *^c^*	**0**.**017***^a^*	**<0.001** *^c^*	**0**.**009***^b^*	**<0.001** *^c^*	0.703
	**AAAB**	0.995	0.714	**<0.001** *^c^*	0.952	0.678	0.999	**0**.**015***^a^*	0.548	**<0.001** *^c^*	**<0.001** *^c^*	0.077	0.170
	**AAB**	0.436	**0**.**027***^a^*	0.315	0.154	0.832	1.000	**0**.**023***^a^*	0.402	**<0.001** *^c^*	**<0.001** *^c^*	0.104	**<0.001** *^c^*
	**ABB**	0.756	1.000	**<0.001** *^c^*	0.957	0.992	0.975	**0**.**041***^a^*	0.793	**0**.**002***^b^*	**<0.001** *^c^*	0.998	**0**.**033***^a^*
**AAA**	**AAAA**	0.840	0.547	0.922	1.000	0.988	1.000	0.490	0.341	0.821	0.983	0.503	0.978
	**AAAB**	0.996	0.999	0.061	0.960	0.282	1.000	0.912	1.000	0.986	0.367	0.109	1.000
	**AAB**	**0**.**009***^b^*	1.000	**0**.**003***^b^*	0.225	1.000	1.000	**0**.**002***^b^*	0.991	**0**.**020***^a^*	1.000	**<0.001** *^c^*	0.998
	**ABB**	0.159	0.330	0.605	0.964	1.000	0.964	0.629	1.000	0.807	0.968	**<0.001** *^c^*	1.000
**AAAA**	**AAAB**	0.986	0.537	0.859	0.981	0.470	0.998	0.237	0.599	0.652	0.967	0.015*^a^*	0.998
	**AAB**	0.990	0.583	**0**.**036***^a^*	0.842	0.989	0.999	**0**.**003***^b^*	0.175	0.998	0.972	**<0.001** *^c^*	0.928
	**ABB**	0.989	0.054	1.000	0.980	0.989	1.000	0.103	0.371	0.378	1.000	**<0.001** *^c^*	0.960
**AAAB**	**AAB**	0.526	0.996	**<0.001** *^c^*	0.986	0.236	1.000	0.703	0.994	0.068	0.282	**<0.001** *^c^*	0.995
	**ABB**	0.667	0.781	0.891	1.000	0.617	0.956	0.999	0.999	0.997	0.917	0.394	0.998
**AAB**	**ABB**	1.000	0.224	**<0.001** *^c^*	0.993	1.000	0.955	0.918	1.000	**0**.**008***^b^*	0.943	0.261	1.000

According to Tukey's multiple range test, significance levels are: *^a^P* < 0.05; *^b^P* < 0.01; *^c^P* < 0.001.

HT, pseudostem height; LBW, leaf blade width; FTH, flowering to harvest; TSS, total soluble solids; TA, titratable acidity; HW, hands weight; BH, number of bunch hands; FL, fruit length; PP, pulp percentage; PDW, pulp dry weight; PT, peel thickness.

**Fig. 1.**
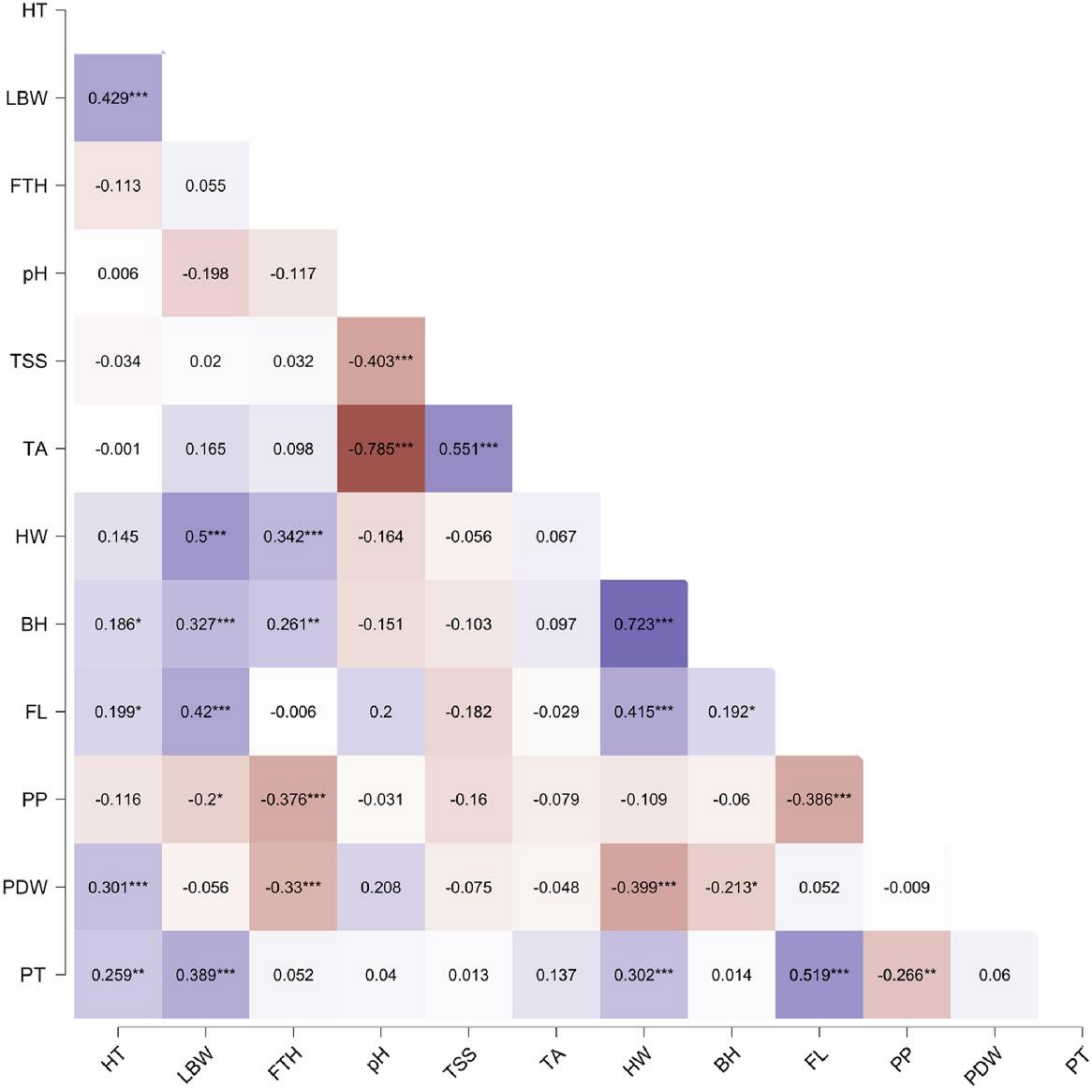
Pearson's correlation coefficient of phenotypic traits evaluated in the *Musa* accessions. HT, pseudostem height; LBW, leaf blade width; FTH, flowering to harvest; TSS, total soluble solids; TA, titratable acidity; HW, hands weight; BH, number of bunch hands; FL, fruit length; PP, pulp percentage; PDW, pulp dry weight; PT, peel thickness. *P < 0.05; **P < 0.01; ***P < 0.001.

Pearson's correlation coefficient analysis allowed us to associate among traits (Fig. 1). The HT had a significant positive correlation with FL (*r* = 0.199, *P* < 0.05) and a highly significant positive correlation with LBW and ST (*P* ≤ 0.01). LBW was significantly correlated with HW, BH, FL, and ST. The trait FTH was positively correlated with BH and HW (*P* ≤ 0.05). In contrast, the variables related to fruit quality were not significantly correlated with the other traits, except for TSS and TA, which were highly significantly positively correlated (*r* = 0.551, *P* < 0.001). Finally, FTH and PDW and FTH and PP were negatively correlated, which may indicate that, at shorter harvest times, it increases the amount of weight and pulp (Fig. 1).

Also, we used a regression model to predict the effect of one or several trait(s) on three of the most critical variables for bananas (HT, TSS, and HW). For HT, model 3 explained 31.4% of the variability, resulting in LBW and PDW as predictor variables. For TSS, the variable TA is the only predictor that explained 36.3% of its variance in model 2. This result is expected since both traits are measures of fruit quality. Finally, model 5 was the best explaining 73.5% variance in HW and the predictor variables were mostly fruit traits (BH, FL, and PDW) and LBW (Table 3).

**Table 3.** Predictive models to determine the possible association between the phenotypic characteristics of *Musa* accessions.

Trait	Model	R	R^2^	RMSE		t	*P*
HT	1	0.000	0.000	0.460	Intercept	45.973	<0.001
	2	0.425	0.180	0.420	Intercept	3.395	0.001
					LBW	3.925	<0.001
	3	0.561	0.314	0.387	Intercept	0.789	0.433
					LBW	3.707	<0.001
					PDW	3.670	<0.001
TSS	1	0.000	0.000	1.302	Intercept	26.885	<0.001
	2	0.602	0.363	1.047	Intercept	13.091	<0.001
					TA	6.312	<0.001
HW	1	0.000	0.000	7509.973	Intercept	14.896	<0.001
	2	0.717	0.515	5269.706	Intercept	−4.691	<0.001
					BH	8.614	<0.001
	3	0.798	0.637	4591.799	Intercept	−6.544	<0.001
					BH	7.707	<0.001
					FL	4.816	<0.001
	4	0.839	0.703	4180.598	Intercept	−1.346	0.183
					BH	7.059	<0.001
					FL	5.716	<0.001
					PDW	−3.904	<0.001
	5	0.857	0.735	3979.299	Intercept	−2.058	0.043
					BH	5.264	<0.001
					FL	3.714	<0.001
					PDW	−4.764	<0.001
					LBW	2.838	0.006

HT, pseudostem height; LBW, leaf blade width; TSS, total soluble solids; TA, titratable acidity; HW, hands weight; BH, number of bunch hands; FL, fruit length; PDW, pulp dry weight.

### PCA and hierarchical clustering based on phenotyping

**Fig. 2.**
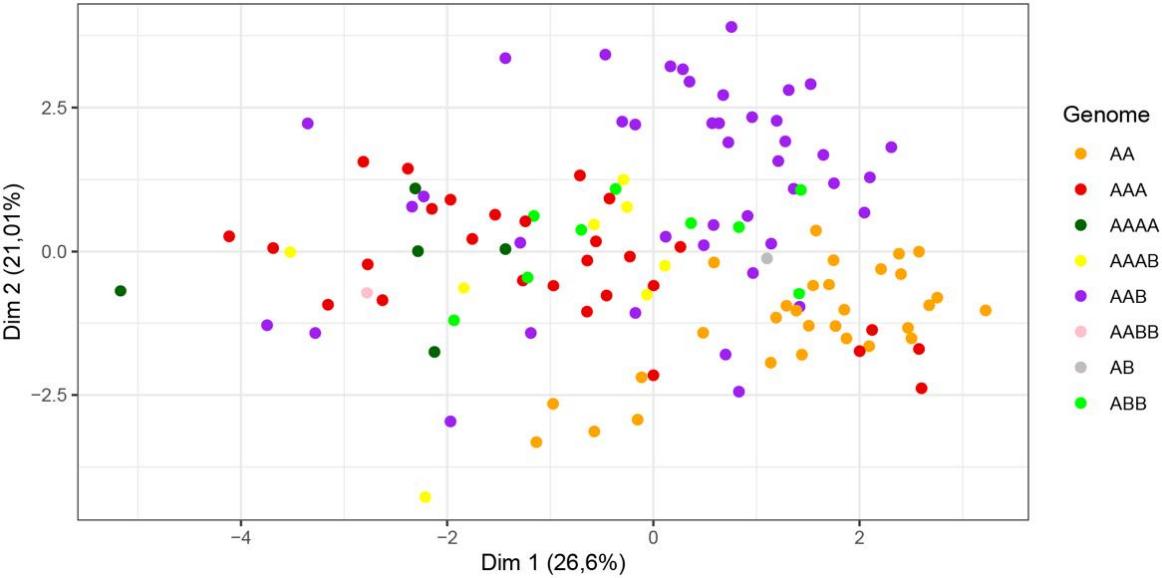
Distribution of *Musa* accessions in the GWAS panel on the first two principal components calculated from phenotypic data.

The first and second principal components (PC1 and PC2) accounted for only 47.6% of total phenotypic variance observed in the GWAS panel (Fig. 2). However, the first four PCs accounted for 69.9% of the total phenotypic variance observed which was above average (Table 4). The main phenotypic factors that contributed to the clustering on PC1 were HW, BH, and LBW in the positive direction and PDW in the negative direction. Similarly, the factors that contributed most to PC2 were TA and TSS in the positive direction and pH in the negative direction. Other factors contributed to PC3 and PC4 in the positive or negative direction (Table 4). Clustering of accessions was mostly influenced by genomic composition although admixture between accessions belonging to different genomic groups was observed.

**Table 4.** PCA based on phenotypic traits evaluated in the *Musa* accessions.

Component	PC1	PC2	PC3	PC4
**Eigenvalue**	3.0111	2.289	1.743	1.309
**Variance (%)**	26.6	21.0	11.8	10.5
**Cumulative variance (%)**	26.6	47.60	59.4	69.90
**Phenotypic trait**				
Hands weight (HW)	0.903			
Number of bunch hands (BH)	0.891			
Leaf blade width (LBW)	0.528			
Pulp dried weight (PDW)	−0.468			0.673
Titratable acidity (TA)		0.918		
pH		−0.865		
Total soluble solids (TSS)		0.751		
Pulp percentage (PP)			−0.899	
Peel thickness (PT)			0.692	
Fruit length (FL)			0.689	
Flowering to harvest (FTH)				−0.775
Pseudostem height (HT)				0.622

**Fig. 3.**
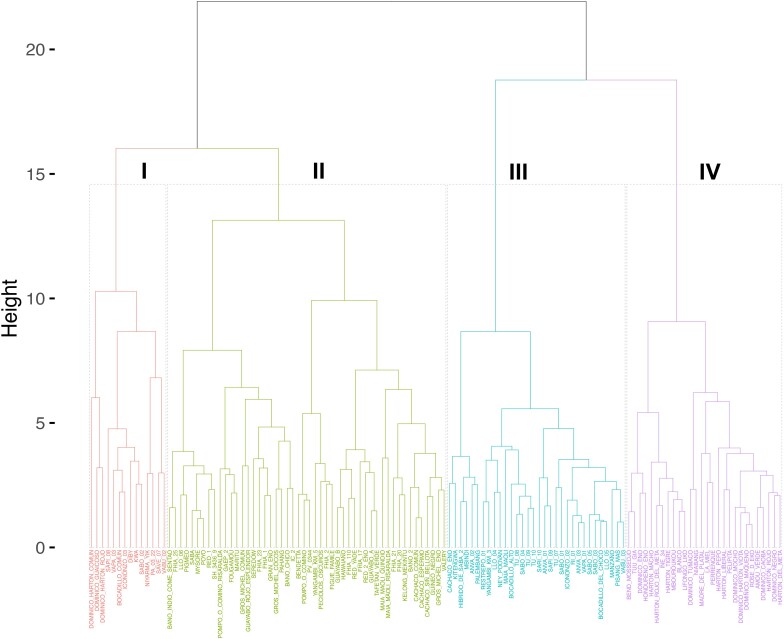
Hierarchical clustering analysis of agricultural traits (morphology, fruit quality, and yield) in 124 *Musa* accessions.

For the hierarchical clustering, plantains and bananas formed distinct groups based on their genomic groups and subgroups. The best value for *K* was 4 to group the 124 *Musa* accessions based on the 12 evaluated traits (Supplementary Fig. 1). Cluster analysis (Fig. 3) showed a first group (I—red color) with 14 accessions, mainly AA diploids (7) and AAB triploid (6), and PA_03_22, an AAAB plantain. The second group (II—green color) contained 50 accessions, mainly grouped by AAA triploid and the AAAA and AAAB tetraploids, and the only AABB tetraploid accession of the collection (GAEP_2). The third group (III—blue color) comprised 32 accessions, mainly with a diploid genome AA (23), in addition to the only AB accession (NEY_POOVAN). The fourth group (IV—pink color) was represented primarily by AAB triploid accessions (26) and two samples with the ABB genome.

### Population structure and LD

The sequence data for each sample had an average depth of ∼7×. We used the original VCF file reported by Higgins *et al*. (2023) to extract the SNPs for the 124 samples used in this study. In total, 187,133 SNPs against *M. acuminata* (A genome) and 220,451 SNPs against *M. balbisiana* (B genome) were identified in the association panel.

**Fig. 4.**
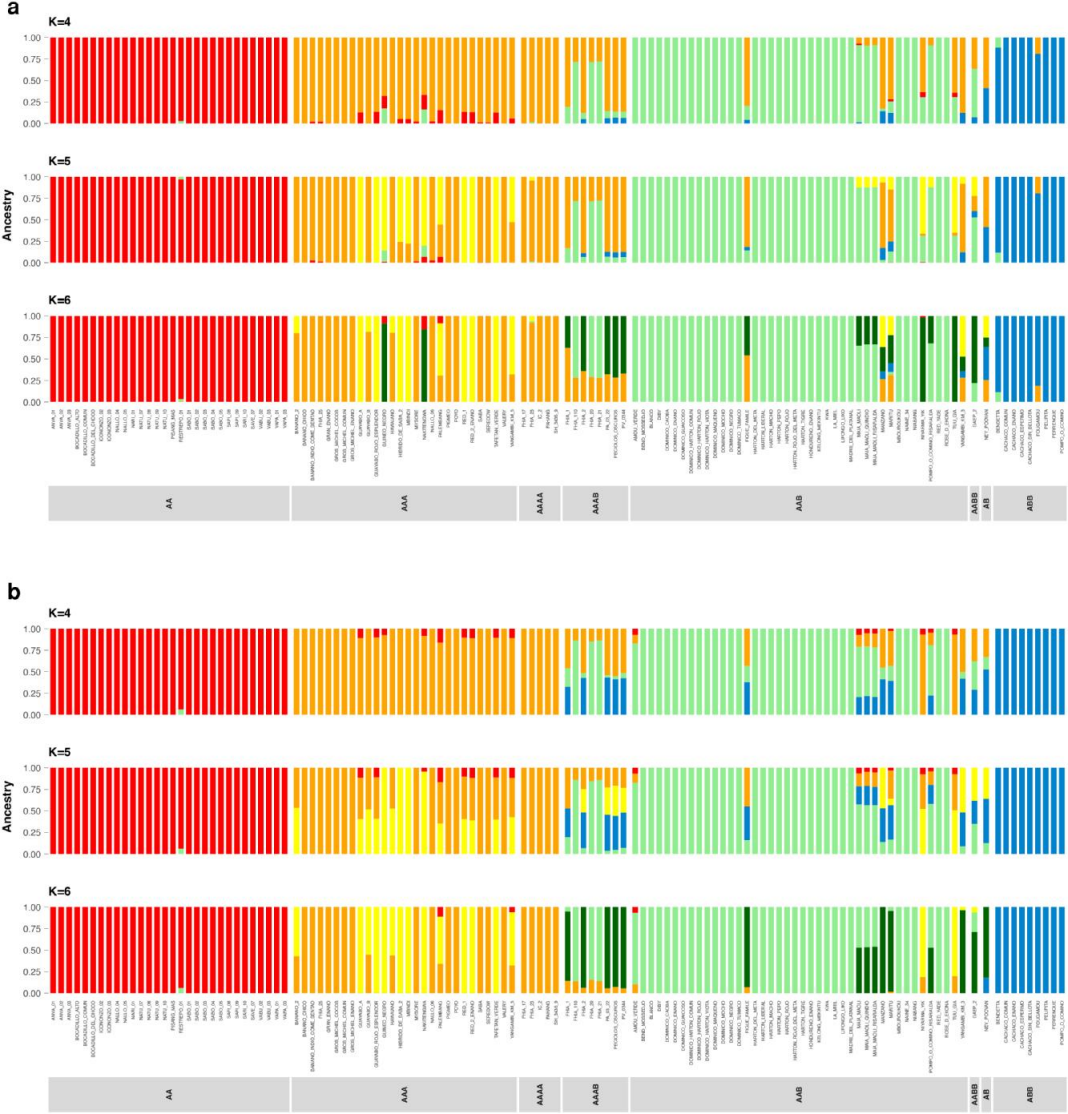
Admixture plot showing the proportion of ancestry shared between *Musa* accessions in the GWAS panel. a) Population structure from SNPs based on *M. acuminata* reference genome, b) population structure from SNPs based on *M. balbisiana* reference genome.

The population structure analysis revealed that 96% of the accessions could be stratified into five populations for the A genome, while the remaining 4% could be regarded as admixtures (Fig. 4a, Supplementary Fig. 2a). For the B genome, 88% of the accessions could be stratified into four populations, while 12% could be considered admixtures (Fig. 4b, Supplementary Fig. 2b). The division into different groups follows the genome composition and ploidy level of accessions. The red group comprised ANVA, Bocadillo, Icononzo, Nallo, Natu, Sabo, and Sapi, among other accessions. The orange group comprised Banano, GrosMichael, and Guayabo accessions, the light-green group principally consisted of Dominico accessions, and the blue group consisted of Cachaco accessions.

In addition, we evaluated the admixture plots for K4, K5, and K6. The *M. acuminata* subspecies were distributed into various clusters. The first cluster regrouped all the Sucrier subgroup accessions (red group in Fig. 4a and b). Diploid accessions AA were highly homogenous, and their ancestry remained restricted to the red group. The diploid accession AB presented a mixed ancestry. From the groups formed by admixture, the accessions from the main banana cultivated subgroups (AA, AAA, AAB, and ABB) presented higher ancestry and were very consistent between genomes and Ks.

When K4 was considered, the orange group formed by AAA and AAAA (Cavendish, GrosMichel) and AAB plantain subgroups, (Fig. 4a and b) presented several samples with unique ancestry for both genomes. However, once K5 and K6 were evaluated, several accessions from AAA presented introgression of new groups (yellow and dark green), such as the accessions Guayabo_A, Guayabo_rojo_esplendor, Guineo negro, Hibrido _de_saba_2, Mbindi, Red_1, and Red_2_enano. A similar situation was presented for some accessions of the AAB genome (light-green group), in which the accessions Figue_Famile, Maia_maoli, Manzano, Maritu, Niyarma_YIK, Pompo_o_comino_Tuu_GIA, and Yangambi_KM_3 presented mixed ancestry. Finally, the blue group comprised homogeneous accessions from the Bluggoe and Pelipita subgroups.

**Fig. 5.**
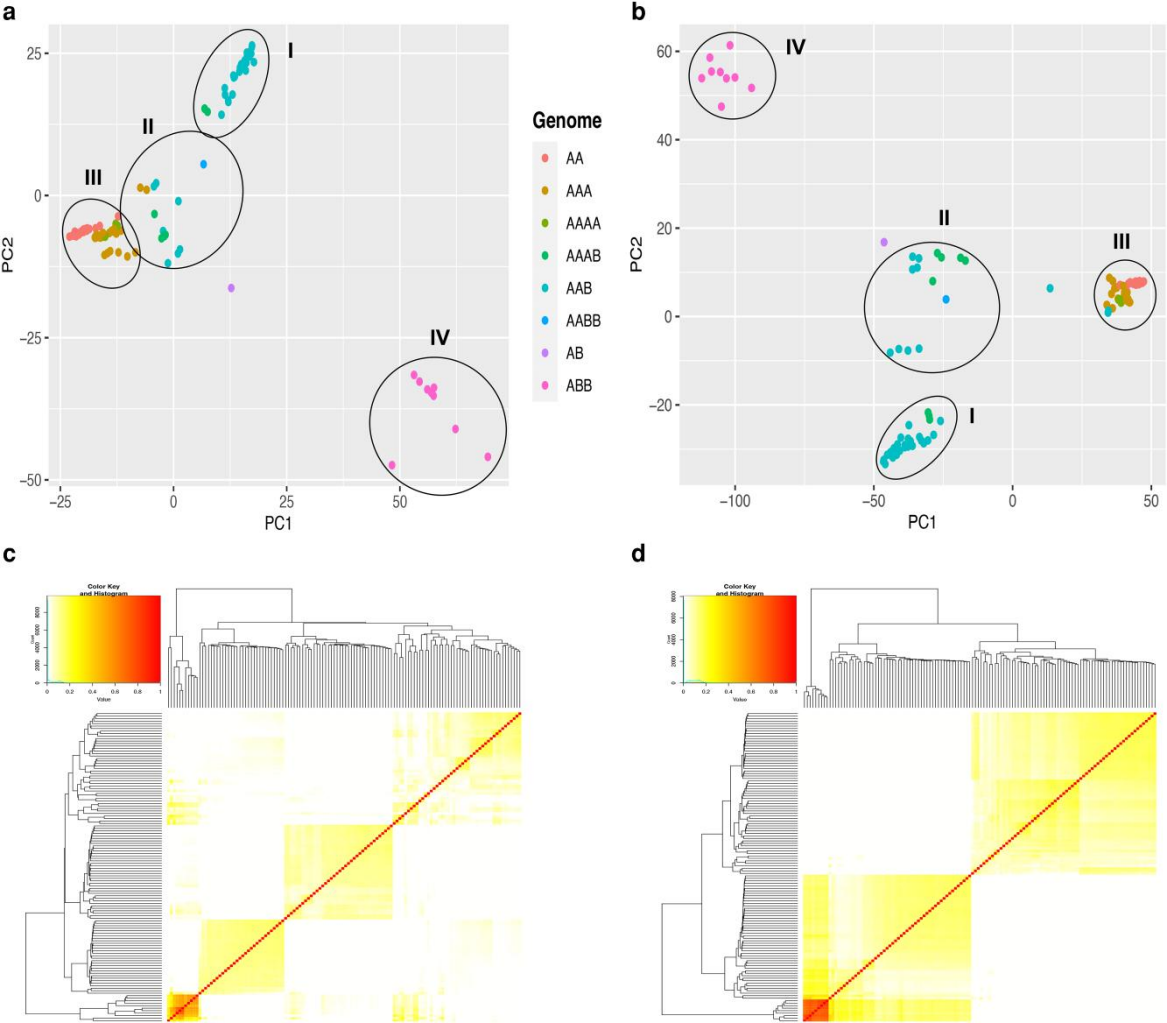
Population structure analysis of the 124 *Musa* accessions. a) PCA based on 187,133 SNPs identified using *M. acuminata* reference genome, b) PCA based on 220,451 SNPs identified using *M. balbisiana* reference genome, c) kinship plot showing the relationship among the genotypes based on 187,133 SNPs, d) kinship plot showing the relationship among the genotypes based on 220,451 SNPs. In the kinship plots, the red and yellow colors represent pairs of individuals with highest and lowest identity, respectively.

Moreover, based on standardized covariance of genetic distances of SNP markers, PCA obtained four clusters for each genome (Fig. 5a and b). The relatedness of pairwise coefficients estimated in the kinship matrix indicated lower genetic relatedness among individuals in the association panel for each genome (Fig. 5c and d).

In the PCAs (Fig. 5a and b), cluster I was composed of most of the accessions of the AAB genome, such as Dominico_caobo and Dominico_enano, and two accessions of the AAAB genome (FHIA_21 and FHIA_110). In cluster II, accessions with AAB and AAAB genomes were regrouped, but Guineo_negro and Nkitenggwa with an “AAA” genome were also included in the PCA of the A genome but not in the PCA of the B genome. Cluster III placed closely most of the accessions that were not presented in the B genome, such as “Cavendish AAA,” “Red AAA,” “Gros Michel AAA,” “Sucrier AA,” and “AAAA.” Cluster IV was conformed uniquely by accessions of the ABB genome. The accessions Ney_poovan and Gaep_2 did not group to any cluster.

The *r^2^* value for the A and B genomes gradually decreased when the genetic distance increased (Supplementary Fig. 3). The mean *r^2^* across the A genome was 0.10 and for the B genome was 0.16. LD decay was 200 kb for the A genome and 100 kb for the B genome, indicating that any SNPs within this distance behave as an inheritance block. This result supported the precise colocalization of MTAs and causative genes within genome blocks using GWAS.

### Marker–trait associations

**Fig. 6.**
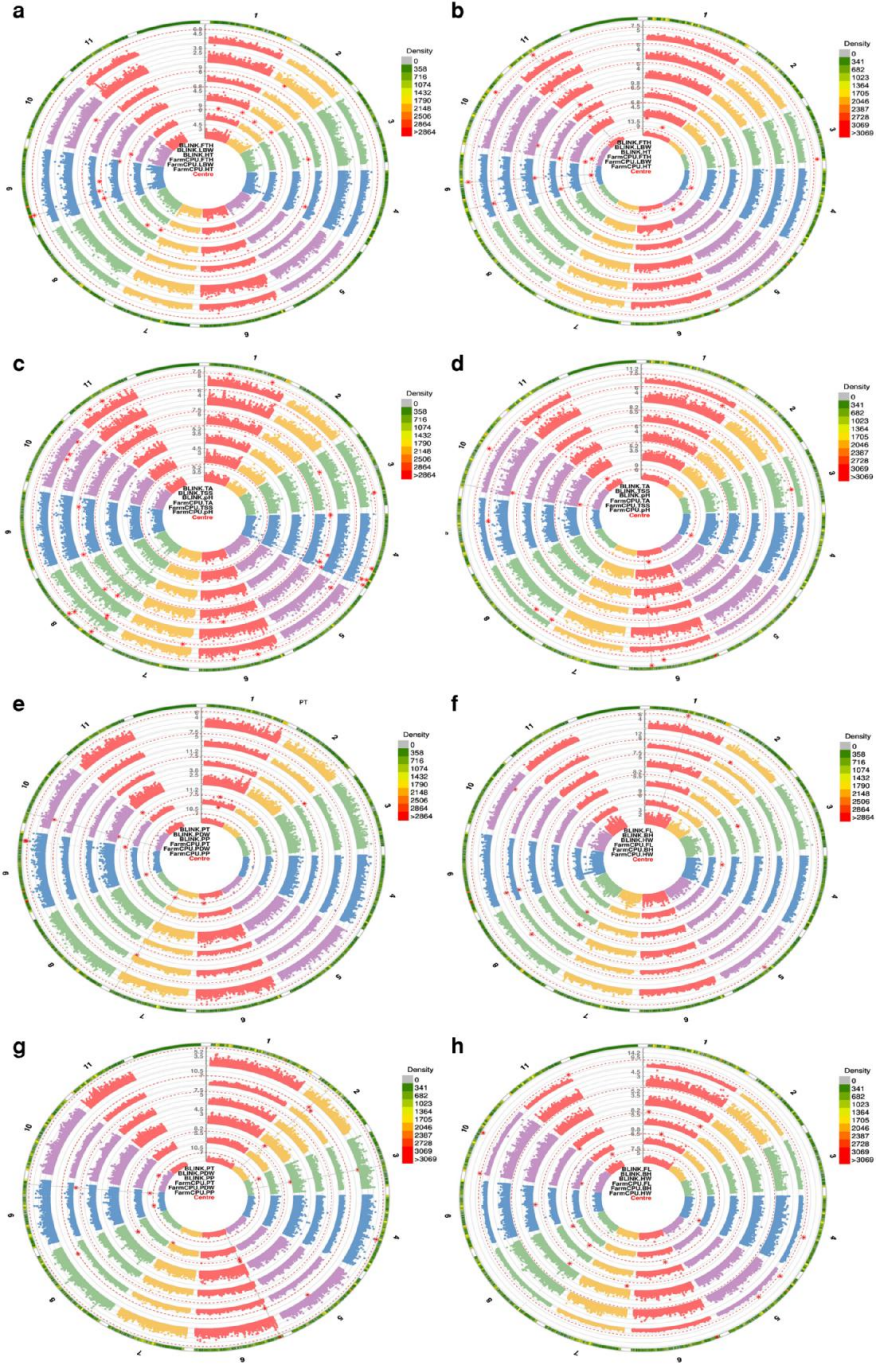
Circular Manhattan plot displaying the chromosome-wide significant market MTAs using BLINK and FarmCPU models. The vertical scale bar represents the significance level of MTAs (−logP values). Individual chromosomes are represented on the outer circle and separated by white borders. Dashed circles indicate FDR thresholds (0.05). Genomic regions of detected QTL on the respective chromosomes are colored in red (outer circle). For plant morphology traits, a) MTA based on 187,133 SNPs identified using *M. acuminata* reference genome, b) MTA based on 220,451 SNPs identified using *M. balbisiana* reference genome. For fruit quality traits, c) MTA based on 187,133 SNPs, d) MTA based on 220,451 SNPs. For yield-related traits (e, f) MTA based on 187,133 SNPs, g, h) MTA based on 220,451 SNPs.

For all the studied traits, 137 MTAs were identified with a significance −log10(*P*-value) over 10. MTAs were filtered by FDR correction value (−log10(*P*) > 5.32) to increase the stringency of selection. Supplementary Tables 3–and 5 show the MTAs above FDR correction, their position in the genome, and the model used for identification. Significant SNPs identified for the studied traits were visualized in Manhattan plots (Fig. 6, Supplementary Fig. 4) and QQ plots (Supplementary Fig. 5).

### MTAs for morphology-related traits

In this category, the marker effect ranged from −36.38 to 39.63, with a PV explained from 0.10 to 50% and a mean value of 15.95%. Fifteen SNPs were found for the A genome associated with morphological traits. Two, five, and eight SNPs were detected for HT, FTH, and LBW, respectively (Fig. 6a, Supplementary Table 4). Using BLINK, the number of significant SNPs identified was one for FTH and two for HT, while FarmCPU identified five SNPs for FTH (one in common with BLINK) and eight for LBW. Finally, the MLM found one SNP for the LBW trait, the same identified using FarmCPU for this genome (Supplementary Fig. 4a). For the B genome, 19 significant SNPs were found for the FTH, HT, and LBW traits (Fig. 6b, Supplementary Table 4). BLINK model found four SNPs for FTH, one for HT, and one for LBW. FarmCPU found three SNPs for FTH (two in common with BLINK), nine for HT, and three for LBW (one in common with BLINK). Finally, MLM identified three SNPs for FTH (one not found by ML-GWAS methods) (Supplementary Fig. 4b). In this category, the S09_47449299 and S09_6752619 SNPs were found for different traits or models in the A genome. Finally, for the B genome, the SNPs S09_21767064, S09_7165470, and S11_12647562 were identified as associated with several traits or models.

### MTAs for fruit quality-related traits

In this category, the marker effect ranged from −4.51 to 4.16, with a PV explained from 7.54e^−9^ to 62.16% and a mean value of 10.17%. The SL-GWAS using the MLM found no SNPs associated with fruit quality traits for either *Musa* genomes (Supplementary Fig. 4c and d). For the A genome, 33 MTAs were found. The BLINK model found 7, 13, and 17 significant SNPs for TSS, PH, and TA, respectively (Fig. 6c, Supplementary Table 5). In addition, the SNPs S04_33402915, S04_40529326, S04_44039837, and S08_9712811 were found for several traits or models. For the B genome, 13 MTAs were identified (Fig. 6d, Supplementary Table 5). The BLINK model identified one, three, and six SNPs for PH, TA, and TSS, respectively. With FarmCPU, four SNPs for PH and one for TA were found. Finally, for the B genome, the SNPs S06_31105046 and S10_2117746 were identified for both models associated with TA and PH, respectively.

### MTAs for yield-related traits

In this category, the marker effect ranged from −8.7 to 7.82, with a PV explained from 0.1 to 73.7% and a mean value of 16.7%. Using the MLM, the SL-GWAS found no SNPs associated with yield-related traits for either of the two *Musa* genomes (Supplementary Fig. 4e–h, Supplementary Table 6). For the A genome, a total of 23 MTAs were found. The FarmCPU model identified two SNPs for BH, three for FL, four for PDW, and four for PP. The BLINK model identified four for BH, two SNPs for FL, one for HW, one for PDW, two for PP, and two for ST (Fig. 6e and f, Supplementary Table 6). Meanwhile, for the B genome, we found 34 MTAs. The FarmCPU model found five SNPs associated with BH (Fig. 6g and h, Supplementary Table 6). Besides, this model found three SNPs associated with HW, five related to FL, and six for PDW. Finally, based on the BLINK model, we found six SNPs for FL, six for PDW, one for PP, and one for ST.

### Putative candidate genes associated with MTAs

Considering *M. acuminata* DH Pahang (v4) as the reference genome, we identified 38 candidate genes within 50-kb regions upstream or downstream of the significant SNPs associated with any trait. In addition, we identified 40 candidate genes for *M. balbisiana* DH-PKW (V1.1). Most identified SNPs were near transcripts coding for proteins or transcription factors. Supplementary Tables 3–4,and 5 list the candidate genes with functional annotation on the NCBI website for each trait category (morphology, fruit quality, and yield). Based on the functional annotations, the most promising candidate genes were related to photosynthesis and metabolism processes, plant hormones, cellular transport, transcriptional regulation, structural proteins, and cell division functions. These genes could directly or indirectly regulate the growth and development of banana plants.

To better characterize the potential consequences of the SNPs, we annotated the variants with SnpEff and predicted the effects of variation on the identified genes (Table 5). A large majority of the variants did not change the amino acid in the protein. For the A genome, three SNPs associated with morphological traits and one with yield traits presented a high effect level, principally related to the loss or gain of a stop codon that could lead to a short polypeptide or elongated transcript. In addition, another effect could be associated with the affectation of alternative splicing by some SNPs. The B genome presented four SNPs associated with morphology, seven SNPs with quality, and six SNPs with yield related to the frameshift variant (sequence variant that disrupts the translational reading frame), splice donor variant (a splice variant that changes the second base pair region at the 5′ end of an intron), intron variant (a transcript variant occurring within an intron), splice acceptor variant (a splice variant that changes the second base region at the 3′ end of an intron), and splice region variant (a sequence variant in which a change has occurred within the region of the splice site, either within 1–3 bases of the exon or 3–8 bases of the intron).

**Table 5.** Effects of significant SNPs predicted by snpEff.

Species	Trait	GWAS Chromosome	SNP GWAS position	SNP SnpEff position	Distance	Effect category	Effect level	Alternative allele count	Alternative allele frequency
***M. acuminata***	Morphology	chr01	12719063	12719292	229	Stop gained	HIGH	101	0.177
			39555304	39555365	61	Stop lost, splice region variant, and conservative inframe deletion	HIGH	8	0.014
				39555366	62	Stop lost and splice region variant	HIGH	31	0.055
				39555367	63	Stop lost and splice region variant	HIGH	99	0.177
		chr09	47449299	47449121	178	Stop gained	HIGH	114	0.200
	Yield	chr07	38033462	38033252	210	Stop gained	HIGH	220	0.388
				38032971	491	Stop gained	HIGH	110	0.194
				38032877	585	Stop gained	HIGH	157	0.278
				38032870	592	Stop gained	HIGH	29–151	0.051–0.268
***M. balbisiana***	Morphology	Bchr03	29586628	29586067	561	Stop lost	HIGH	469	0.823
				29586700	72	Stop lost	HIGH	47	0.083
				29587052	424	Stop lost	HIGH	456	0.809
				29587055	427	Stop gained	HIGH	455	0.807
				29587097	469	Stop lost	HIGH	54	0.096
				29587103	475	Stop gained	HIGH	20	0.036
				29587256	628	Stop lost	HIGH	449	0.792
		Bchr05	34530451	34530629	178	Frameshift variant	HIGH	19	0.033
				34530630	179	Frameshift variant	HIGH	22–11	0.039–0.019
				34530666	215	Stop lost	HIGH	26	0.046
				34530673	222	Frameshift variant	HIGH	25	0.044
				34530674	223	Frameshift variant	HIGH	16	0.028
				34530678	227	Stop gained	HIGH	9–56	0.016–0.098
				34530688	237	Frameshift variant	HIGH	9	0.016
				34530693	242	Splice donor variant and intron variant	HIGH	18	0.032
				34530694	243	Splice donor variant and intron variant	HIGH	18	0.032
				34530822	371	Splice donor variant and intron variant	HIGH	62	0.109
				34530832	381	Frameshift variant and stop gained	HIGH	277	0.489
				34530863	412	Frameshift variant	HIGH	248	0.440
				34530864	413	Frameshift variant	HIGH	246–248	0.434–0.437
				34530866	415	Frameshift variant	HIGH	487	0.863
		Bchr06	33091399	33091574	175	Stop lost and disruptive inframe deletion	HIGH	133–321	0.237–0.572
				33091582	183	Stop lost	HIGH	359	0.643
		Bchr10	32201530	32200795	735	Splice acceptor variant and intron variant	HIGH	32	0.056
				32200884	646	Stop gained	HIGH	11–2	0.019–3.527e-03
	Quality	Bchr04	38049326	38049086	240	Frameshift variant	HIGH	372	0.656
				38049402	76	Stop lost	HIGH	276	0.487
				38049530	204	Frameshift variant	HIGH	104	0.183
				38049531	205	Frameshift variant and splice region variant	HIGH	253–90	0.446–0.159
		Bchr08	10478815	10478050	765	Stop gained	HIGH	48–12	0.085–0.021
				10478074	741	Frameshift variant	HIGH	15	0.026
			10678591	10677986	605	Stop gained and splice region variant	HIGH	47	0.193
	Yield	Bchr01	17193812	17193320	492	Stop lost	HIGH	479	0.840
				17193325	487	Stop gained	HIGH	117	0.205
				17193330	482	Stop lost	HIGH	25	0.044
				17193490	322	Frameshift variant	HIGH	265	0.465
				17194030	218	Stop lost	HIGH	62	0.109
		Bchr06	1508813	1508438	375	Stop gained	HIGH	303–191	0.561–0.354
				1508513	300	Stop gained	HIGH	245	0.432
				1508572	241	Frameshift variant	HIGH	33	0.058
				1508576	237	Stop lost	HIGH	16–35	0.028–0.062
				1508759	54	Stop lost	HIGH	305	0.538
				1508764	49	Stop lost	HIGH	108	0.190
				1509011	198	Frameshift variant	HIGH	17	0.030
				1509016	203	Frameshift variant	HIGH	149,6	0.266–0.011
				1509028	215	Stop gained	HIGH	35	0.062
				1509037	224	Stop lost	HIGH	375–143	0.668–0.255
			12662938	12663145	207	Stop gained	HIGH	174	0.305
		Bchr07	34950115	34949803	312	Splice acceptor variant and intron variant	HIGH	285	0.503
				34949981	134	Frameshift variant	HIGH	6	0.011
		Bchr08	38003235	38002633	602	Stop gained	HIGH	46	0.081
				38002869	366	Stop lost	HIGH	6	0.011
			40937646	40937984	338	Stop lost	HIGH	192	0.340
				40937989	343	Frameshift variant	HIGH	179–78	0.316–0.138
				40938023	377	Splice acceptor variant and intron variant	HIGH	473	0.834
				40938126	480	Stop gained	HIGH	472	0.837
				40937984	338	Stop lost	HIGH	192	0.340
				40937989	343	Frameshift variant	HIGH	179–78	0.316–0.138
				40938023	377	Splice acceptor variant and intron variant	HIGH	473	0.834
				40938126	480	Stop gained	HIGH	472	0.837

## Discussion

Banana producers look for specific morphology, fruit quality, and yield features, which influence the adoption of new banana varieties. Identifying the genomic regions controlling these agronomic traits is a fast and intelligent way to generate knowledge for developing new varieties with desirable features. Genome-wide SNP markers with a deep phenotypic characterization can assist breeders in dispensing higher genetic gains (Mammadov *et al.* 2012; Morgil *et al.* 2020). In the present study, we conducted a GWAS based on genetic and phenotypic information in banana accessions conserved in the AGROSAVIA germplasm. We aim to improve our knowledge of genetic architecture and the inheritance of crucial agronomic banana traits.

We measured 12 traits describing morphology, fruit quality, and yield in 124 banana accessions. Most traits followed a normal distribution, supporting their relative stability. Standard deviations (SDs) revealed significant variation, indicating the data validity for statistical analyses. The SD was low for HT, pH, TSS, TA, BH, and ST. Instead, LBW, HW, FTH, PDW, PP, and FL had high SD, indicating increased variability of these characteristics among the accessions. This variability is due to the presence of genomic groups with different subgenome compositions, which in turn are composed of different clonal varietal subgroups, such as Gros Michel, Cavendish, Red, Lujugira/Mutika, Sucrier, Plantain, Bluggoe, Popoulu, and Pelipita. Likewise, it is important to highlight the significant differences between HW, FL, and PDW in the different subgenome compositions. These descriptors have been highlighted for their discriminating power between cultivars according to their genomic composition and A and B genome contributions. Unlike AA and AAA bananas, the cultivars with the highest FL and PDW are related to the genomic composition with the highest *M. balbisiana* contribution, such as AAB and ABB (Dufour *et al.* 2009).

It is essential to highlight the significant positive correlations between HT and LBW, FL, and ST, as well as between LBW and HW, BH, FL, and ST. These correlations indicate that taller plants have larger leaves that provide the transformation capacity of photoassimilates, forming larger fruit clusters with a high amount of pulp, which is an essential feature for crop yield. The PCA showed that yield traits explained most of the variability in our collection, probably due to the banana domestication process, and yield was of great importance for the farmers. Larger fruits are positively correlated with the presence of thicker peels (PT). Accessions with this characteristic may be interesting for the cellulose fiber industry, which uses banana peels as a biomaterial for paper production (Khawas *et al.* 2016). Likewise, FTH is positively correlated with BH and HW, which may indicate that, at longer harvest times, there will be a higher number of hands and weight.

Regarding hierarchical clustering, the groups were differentiated by their genetic variability. AA diploid and AAB triploid bananas formed the first cluster with a distinguished phenotype (Fig. 3). These bananas tend to be consumed fresh or directly (Dufour *et al.* 2009) due to their pulp characteristics and high sugar content when ripe, consistent with the higher values of pH, TSS, and low organic acid (AT) contents observed. These traits are essential indicators of fruit quality, according to Enriquez Valencia (2021), who evaluated the physicochemical properties of flours and starches of AA diploid and AAA triploid materials, highlighting their importance for fresh consumption. Diploid bananas from cluster one differed from those in the third cluster in variables associated with bunch size, such as FL, PP, and ST. The Sucrier cultivars (AA) are characterized by small fruits with a high PP and thinner peels. Enriquez Valencia (2021) found significant differences between the physical characteristics (bunch and fruit size and the number of hands and fingers per hand) of diploid bananas from clusters one and three.

The second cluster regrouped accessions with low dry matter content (PDW) and fresh consumption use (Gibert *et al.* 2009), especially the Gros Michel and Cavendish subgroups. The accessions regrouped in the fourth cluster presented characteristics related to the contribution from the B genome. These accessions are adapted for the frying industry due to the high PDW content that improves oil absorption (Dufour *et al.* 2009; Gibert *et al.* 2009). Likewise, these accessions can be used by the paper industry using rind cellulose, as they contain a thicker rind and (ST) and larger fruits (FL) (Silva *et al.* 2001).

Admixture analysis and the PCA presented similarities in the obtained groups. Both analyses differentiated the ABB accessions and a large part of the AAB individuals. However, the PCA failed to separate the AA, AAA, and AAAB genomes in different clusters compared to the admixture analysis. Phenotypic and genetic data produced dissimilar groups. Genetic analyses clustered accessions with similar genome types, while the phenotype analyses formed clusters with mixtures of different genomes.

To study the genetic inheritance of the described traits by GWAS, we conducted an extensive genomic characterization of the same germplasm using the skim-seq method (Kumar *et al.* 2021; Adhikari *et al.* 2022). Rare alleles sometimes control the variation of phenotypic traits, but identifying rare variants through GWAS is challenging and requires high diversity and robust phenotypic evaluation (Tibbs Cortes *et al.* 2021). The methodology used in this study provides comprehensive sequencing that detects even rare alleles with high confidence levels. GWAS analysis may produce false associations because it can be affected by the population structure and the inclusion of diverse genotypes. Our study presented an advantage in comparison with other GWAS studies in *Musa* because in our panel we included diploids, triploids, and tetraploids genotypes belonging to 13 varietal genetic clusters, wild and single representatives of other common cultivars (Higgins *et al.* 2023). The marker density produced was higher than in other studies (Sardos *et al.* 2016; Nyine *et al.* 2019), which allowed us to comprehensively analyze the population structure and carry out a GWAS with good resolution.

The number of markers in a determined genetic distance required for association mapping is determined by the extent of LD decay (Flint-Garcia *et al.* 2003). As expected in an outcrossing species, the analyses demonstrated a rapid decline in LD. The prevailing characteristics of outcrossing species, including high recombination and mutation rates and gene conversion (Sorkheh *et al.* 2008), explain this LD behavior. Compared to findings from other studies, our LD analysis results revealed a relatively lower mean *r^2^* value (0.10–0.16) than the reported value of 0.25 (Sardos *et al.* 2016) but equivalent to the mean *r^2^* = 0.15 reported by Nyine *et al*. (2019). This outcome is explained by the high genetic diversity in our dataset. Flint-Garcia (2013) stated that the admixture between individuals of genetically distinct populations leads to the rapidly decreasing LD of different ancestries. High genetic diversity and rapid LD decay are possibly a reflection of the intra- and inter-specific hybrid origin of studied accessions, resulting from outcrossing between seed-bearing subspecies of *M. acuminata* and *M. balbisiana* comparable to other nondomesticated crop species such as *Chenopodium quinoa* Willd (Patiranage *et al.* 2022). Although *Musa's* selection began around 7,000 years ago (Denham *et al.* 2003), this crop has been cultivated via vegetative propagation. Endeavors to generate novel varieties through hybridization, mutation, or transformation have encountered challenges caused by species’ genetics and sterility (Heslop-Harrison and Schwarzacher 2007).

Mir *et al*. (2021) defined yield as a very complex quantitative trait controlled by a network of many small-effect minor genes or QTLs. It is a challenge to apply GWAS to study complex traits resulting from the cumulative effect of QTLs, epistasis (interactions between QTLs), and the interaction between environment factors and QTL (Andrade *et al.* 2020; Merrick *et al.* 2022). For such polygenic traits, it is necessary to sample a large population with phenotypic diversity to improve the detection of meaningful associations. Using GWAS, Nyine *et al*. (2019), using a dataset of 307 genotypes and 27,178 SNPs, identified QTL in the *Musa* AAA group for productivity-related traits, such as fruit number. They identified 25 genomic loci, primarily localized on chromosome 3, and concluded that a few QTLs with major effects controlled yield in the studied population. Sardos *et al*. (2016) detected 13 candidate genomic regions potentially linked with the seedless phenotype (i.e. parthenocarpy combined with female sterility) using a panel of 105 accessions of *M. acuminata* and 5,544 SNPs from GBS data. Hence, our GWAS is robust because it included 124 accessions of two *Musa* species (*M. acuminata* and *M. balbisiana*) and over 150 K SNPs to perform comparative models, such as SL-GWAS and ML-GWAS.

The QQ plot of *P*-values comparing observed and expected (random) exhibited a diagonal linear shape, confirming the models’ power in discerning genuine MTAs. In this study, ML-GWAS models outperformed SL-GWAS, which has been reported in several plant studies (Kaler *et al.* 2020; Zhong *et al.* 2021; Adhikari *et al.* 2023). Based on our results, summarizing all the traits, the BLINK performed better than FarmCPU because it found more MTAs, possibly due to its higher computational power.

We identified 22 genes related to morphological traits in 34 loci (15 for the A genome and 19 for the B genome). In contrast, MLM found only a single gene undetected by the ML-GWAS models. In addition, for this category FarmCPU found more MTAs than BLINK. Several genes identified by ML-GWAS merit attention for their role in regulating plant growth and development, such as the GSTs (Jiang *et al.* 2010) on chromosome 1 (S01_12719063), bric-à-brac, Tramtrack, broad (BTB) gene (Chevrier *et al.* 2014) located on chromosomes 2 and 9 (S02_10779672 and S09_24200380, respectively), and polygalacturonases located on chromosome 3 (S03_36192969) (Yang *et al.* 2018). The gene reversionless1 (*REV1*) (S10_26700057) could be essential for tolerance to stress because it plays a role in DNA damage tolerance and repair (Schröpfer *et al.* 2014). The branched-chain amino acids found on chromosome 10 (S10_6660970) are related to catabolism genes in stress, development, and the diurnal/circadian cycle (Peng *et al.* 2015).

The three models identified the association of FTH with the SNP S09_21767064 in chromosome 9 close to the gene NADH ubiquinone (UQ) oxidoreductase (complex I), which provides the input to the respiratory chain from the NAD-linked dehydrogenases of the citric acid cycle (Kerscher 2000). The SNP S09_7165470 on chromosome 9 was associated with LBW, which is close to the jacalin-related lectin gene that has a signaling response to multiple stresses (Song *et al.* 2014) and in plant secondary metabolism (Hosmani *et al.* 2013). Finally, we found two SNPs (S10_8799377 and S10_8799377) in chromosome 10 associated with FTH and HT, respectively, related to cytochrome *c* oxidase, an electron acceptor of the respiratory chain, involved in the reduction of O_2_ to H_2_O (Mansilla *et al.* 2018). Two SNPs in chromosome 11 were located in the polyprenyl diphosphate synthase gene, which plays essential roles in the biosynthesis of functionally important plastoquinone and UQ, involved in electron transfer and energy transformation in the plastids and mitochondria (Liu *et al.* 2019). The SNP S10_1387129 in chromosome 10 was also found close to transcription factor MYB60 involved with stomatal opening and reported as a transcriptional integrator of oxylipins responses in guard cells and abscisic acid in *Arabidopsis thaliana.* This transcription factor induces the closure of stomatal pores to reduce water loss by transpiration (Rusconi *et al.* 2013). Other genes associated with morphological traits belong to a few functional groups, such as membrane vesicle trafficking, transcriptional regulation, redox, and cellular transports, which are critical for plant growth and development.

In the fruit quality category, PH, TA, and TSS of *Musa* were associated with multiple genes according to our GWAS results. Improving crop yield presents a challenge as it can potentially compromise quality, a well-known phenomenon with implications for crop breeding. This challenge arises from the inherent negative correlation between yield and quality traits (Wallace *et al.* 2018). Our study diverged from this, as we identified no substantial correlation between fruit quality and the other yield-related features. However, the accessions we studied exhibited a notable range of variability and modest yields, differing from the high-yielding genotypes explored in previous research (Wallace *et al.* 2018). Fruit maturity and quality are tied to TSS concentration and TA content (Subedi and Walsh 2011; Youryon and Supapvanich 2017). For instance, the softening of banana fruit can be attributed to the degradation of cell wall compounds, a decrease in starch content, and augmentation in sugar levels (Li Wen *et al.* 2006), while the variation in TA content, on the other hand, is influenced by the genomic composition (Youryon and Supapvanich 2017).

Of particular interest, genes associated with TA or TSS traits have emerged from our analysis. Notably, the SNP S04_41216210 identified on chromosome 4 is linked to WRKYs, which hold significance due to their involvement in biotic/abiotic stress responses and developmental and physiological processes (Phukan *et al.* 2016). On chromosome 6, we found three SNPs (S06_12286050, S06_12286054, and S06_12286057) related to the gene mevalonate diphosphate decarboxylase. This enzyme catalyzes the decarboxylation of six-carbon MVA-PP to five-carbon isopentenyl diphosphate, a fundamental structure required for isoprenoid biosynthesis that is a vital cellular intermediate (Krepkiy and Miziorko 2004). Another notable finding is the SNP S10_20397479 located in the transcription factor MYB59, which is crucial in regulating cell cycle progression and root elongation in *A. thaliana* (Fasani *et al.* 2019). While our study sheds light on genes implicated in *Musa* fruit quality, further research is imperative to unravel the precise functions of these genes.

For the yield traits, 57 MTAs were found. Most SNPs were located in genes encoding conserved and hypothetical proteins, but some mapped to known transcription factors and genes involved in diverse cellular processes. Nyine *et al*. (2019) reported 25 significant QTLs, primarily localized on chromosome 3. In contrast, our investigation discovered novel robust association signals on distinct chromosomal regions. For the A genome, we identified six SNPs on chromosome 9 and four SNPs on chromosome 1, while for the B genome, we identified four SNPs on chromosomes 6, 8, and 10. According to the outcomes of our GWAS analysis, it is evident multiple genes would control yield-associated traits.

As expected, the identified SNPs that had a high effect on gene function were found in a smaller proportion. Further experimental verification is necessary to confirm that predicted impacts are as follows: (1) widespread in the population or if it is present in only one individual and (2) if they produce or affect the phenotype. On the other hand, heritability is a population parameter that measures the degree of variation in a phenotypic trait due to genetic variation (Schmidt *et al.* 2019). Therefore, it is reasonable to expect a positive relation between heritability and the ability to detect associations. In the current population, high h^2^_bs_ was found for the traits, varying from 0.25 for TA to 0.99 for PDW, suggesting that the phenotypic variations of all traits are mainly affected by genetic factors. In line with this, most of the individual markers explained a small portion of the phenotypic variation (from 10% for quality traits to 16.7% for yield traits), with just a few markers that explained >30%. Similar high *H*^2^ results were reported by Nyine *et al*. (2019), who found that bunch weight was 0.92, the number of hands was 0.88, the number of fruits was 0.83, and the fruit length was 0.9. Our finding diverges from the report by Nyine *et al*. (2019), who postulated that few QTLs with major effects govern the yield expression. Our study reveals contrasting results, suggesting that the phenotypic variation depends on the cumulative actions of many genes with minor effects, potentially attributed to evaluating different *Musa* populations, using two distinct reference genomes, and employing three analytical models.

Our investigation offers novel insights into the underlying genetics governing morphology, fruit quality, and yield traits, laying a solid foundation for comprehensive functional studies. The markers identified through our rigorous analysis are promising for pyramiding favorable alleles in new cultivars embodying a desirable suite of traits. The stable SNPs identified by different models and related to various characteristics are of particular significance, underscoring their robustness and applicability. It has been suggested that it is preferable to have populations with 100–500 individuals to have robust results in GWAS (Kumar *et al.* 2012). Our study had a population of 124 samples from which high *P*-values were obtained. In further studies in *Musa*, a suitable improvement could be to increase the population size to have a good representation of different ploidy levels and thus to improve the detection power of meaningful associations with large effects. In the close future, we will confirm the MTAs identified, validating the presence/absence of the SNP associated with the trait using competitive allele-specific PCR markers. This transformation will enable the assessment of their efficacy in pinpointing individuals possessing the desired traits within bi-parental populations. Our study holds the potential to help *Musa* improvement strategies through informed and targeted breeding approaches.

### Conclusions

In the current investigation, we harnessed the power of GWAS methodologies, conducting a comprehensive analysis involving 124 *Musa* accessions. This rigorous approach revealed a compendium of SNP markers and prospective candidate genes exhibiting significant associations with 12 agronomic traits. This work contributes theoretical depth and practical utility to genetic breeding, particularly concerning traits relevant to *Musa* collections. Furthermore, it accentuates the efficacy of employing ML-GWAS models, a dynamic tool that effectively pinpoints many MTAs within complex traits. Our findings supported the precision and effectiveness of trait-focused genetic evaluation within *Musa* species, providing a solid footing for future advancements in crop improvement strategies.

## Supplementary Material

jkae108_Supplementary_Data

## Data Availability

Germplasm is held in AGROSAVIA's collection (MGIS: COL004) and available on request. The datasets presented in this study can be found in online repositories. The names of the repository/repositories and accession number(s) can be found below PRJEB62882 in the European Nucleotide Archive. Plant accessions were obtained from AGROSAVIA's gene bank in compliance with national laws and international treaties. Supplemental material available at G3 online.
