# Supplementary material for: Genome-wide association analyses using multilocus models on bananas (Musa spp.) reveal candidate genes related to morphology, fruit quality, and yield
Source: G3 (Bethesda). 2024 May 22;14(8):jkae108. doi: 10.1093/g3journal/jkae108 (PMC11304972; doi:10.1093/g3journal/jkae108)
Supplement: jkae108_Supplementary_Data [file jkae108_supplementary_data.zip › Supplemental_Material_Legends_G3-2024-405047.docx]

**SUPPLEMENTAL DATA**

**Table S1.** A panel of 124 Musa accessions used in the genome-wide association study.

**Table S2.** Phenotypic datasets presented in this study.

**Table S3.** Descriptive statistics for traits evaluated in the GWAS panel.

**Table S4.** Significant marker-trait associations with a p-value corrected by FDR for fruit morphology-related traits for each model and putative candidate genes identified at 50 kb.

**Table S5.** Significant marker-trait associations with a *p*-value corrected by FDR for fruit quality-related traits for each model and putative candidate genes identified at 50 kb.

**Table S6.** Significant marker-trait associations with a *p*-value corrected by FDR for yield-related traits for each model and putative candidate genes identified at the 50 kb region of Linked SNPs along with their molecular functions.

**Figure S1.** The optimal number of clusters for hierarchical cluster analysis for 124 *Musa* accessions. The analysis showed the best *K* at 4.

**Figure S2.** Plot of ADMIXTURE cross-validation error from K=1 through K=10 to analyze the SNP data as the value that minimizes the error. (A) *M. acuminata* shows the lowest error and best *K* at 5, (B) *M. balbisian*a genome shows the lowest error and best *K* at 4.

**Figure S3.** Plots modeling the decay in pairwise linkage disequilibrium (LD) decay in a GWAS panel of 124 diverse *Musa* accessions. The LD was calculated using an *r*^2^ coefficient as a function of the distance between markers in kilobases (Mb) using the SNPs found against two reference genomes. (A) LD decay for *M. Acuminata*. (B) LD decay for *M. balbisiana*.

**Figure S4.** Circular Manhattan plot displaying the chromosome-wide market trait associations (MTA) for yield-related traits using MLM. The vertical scale bar represents the significance level of marker-trait associations (−logP values). Individual chromosomes are represented on the outer circle and separated by white borders. Dashed circles indicate FDR thresholds (0.05). Genomic regions of detected QTL on the respective chromosomes are colored in red (outer circle). For plant morphology traits, (A) MTA based on 187,133 SNPs identified using *M. acuminata* reference genome, (B) MTA based on 220,451 SNPs identified using *M. balbisiana* reference genome. For fruit quality traits, (C) MTA based on 187,133 SNPs, (D) MTA based on 220,451 SNPs. For yield-related traits (E) (F) MTA based on 187,133 SNPs, (G) (H) MTA based on 220,451 SNPs*.*

**Figure S5.** Quantile–quantile plots of estimated−log10 (*P*) from genome-wide association studies obtained with GAPIT. (A) Q-Q plots based on 187,133 SNPs identified using *M. acuminata* reference genome, (B) Q-Q plots based on 220,451 SNPs identified using *M. balbisiana* reference genome. The plots provide no evidence of bias in the GWAS, such as due to genotyping artifacts, and display the extent to which the observed distribution of the test statistic followed the expected (null) distribution. The red line represents expected *p*-values with no associations.
