# Supplementary figures and images for: Genome-wide association analyses using multilocus models on bananas (Musa spp.) reveal candidate genes related to morphology, fruit quality, and yield
Source: G3 (Bethesda). 2024 May 22;14(8):jkae108. doi: 10.1093/g3journal/jkae108 (PMC11304972; doi:10.1093/g3journal/jkae108)

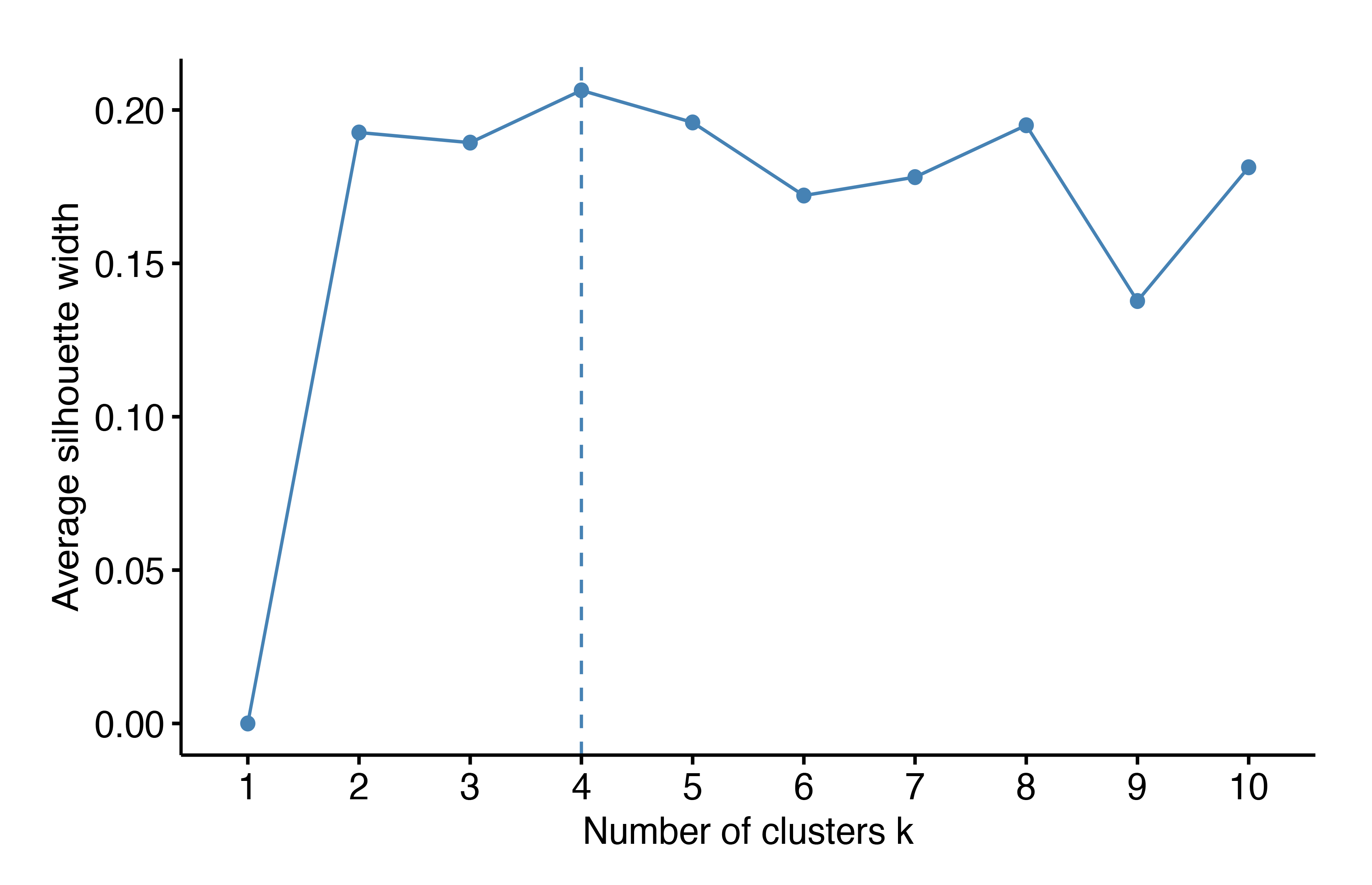

Supplement: jkae108_Supplementary_Data [file jkae108_supplementary_data.zip › Figure_S1_G3-2024-405047.tif]

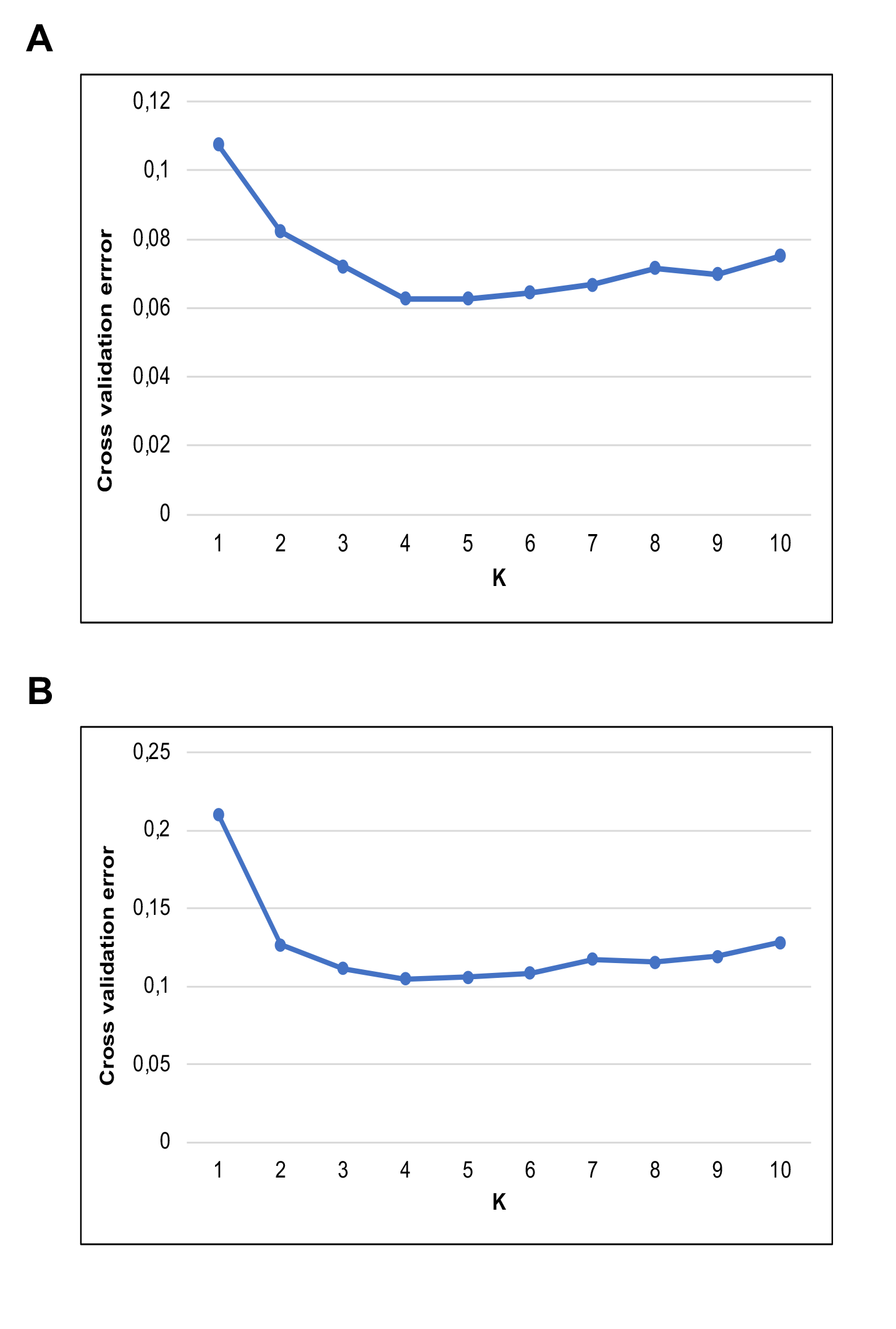

Supplement: jkae108_Supplementary_Data [file jkae108_supplementary_data.zip › Figure_S2_G3-2024-405047.tif]

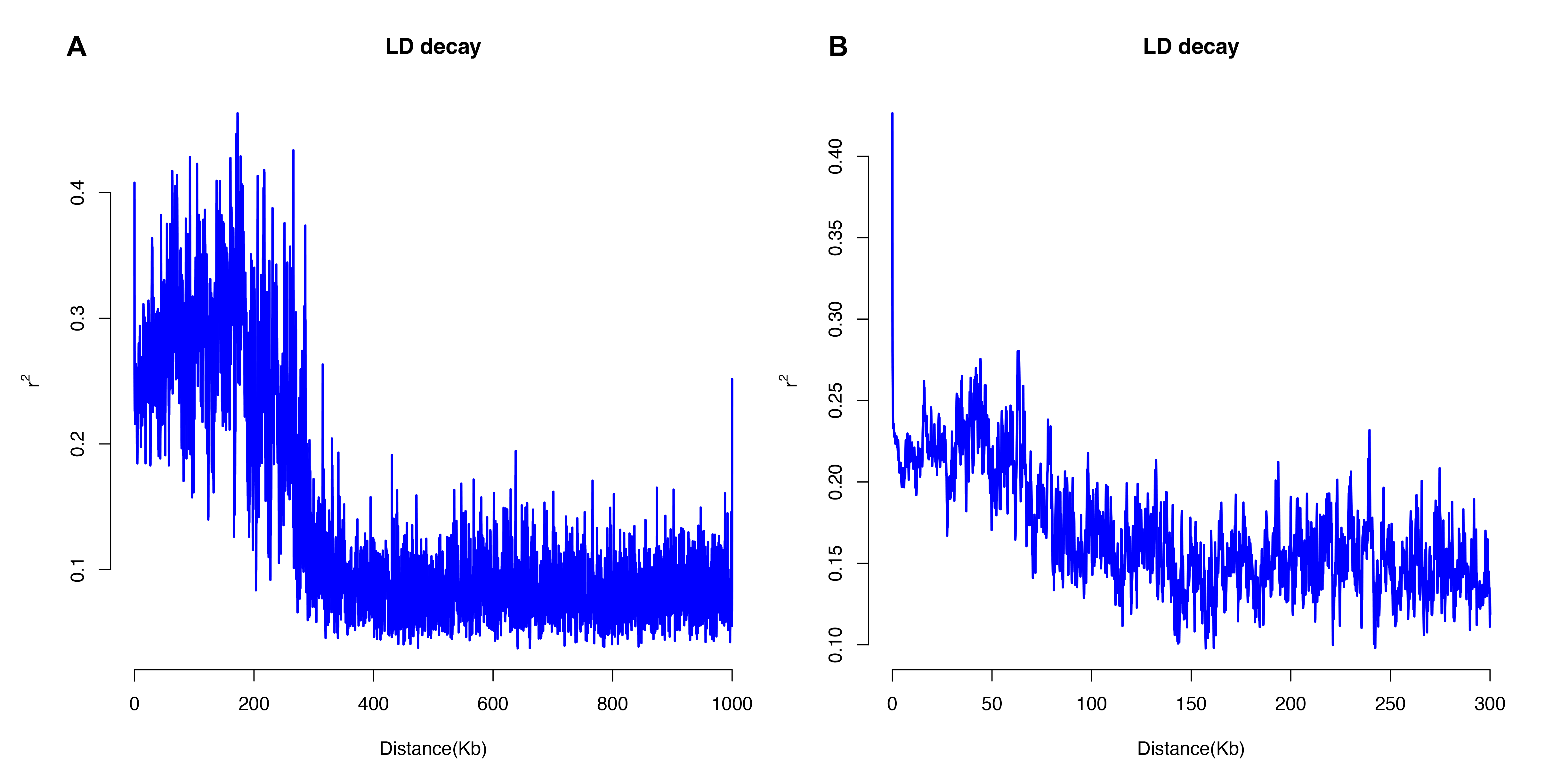

Supplement: jkae108_Supplementary_Data [file jkae108_supplementary_data.zip › Figure_S3_G3-2024-405047.tif]

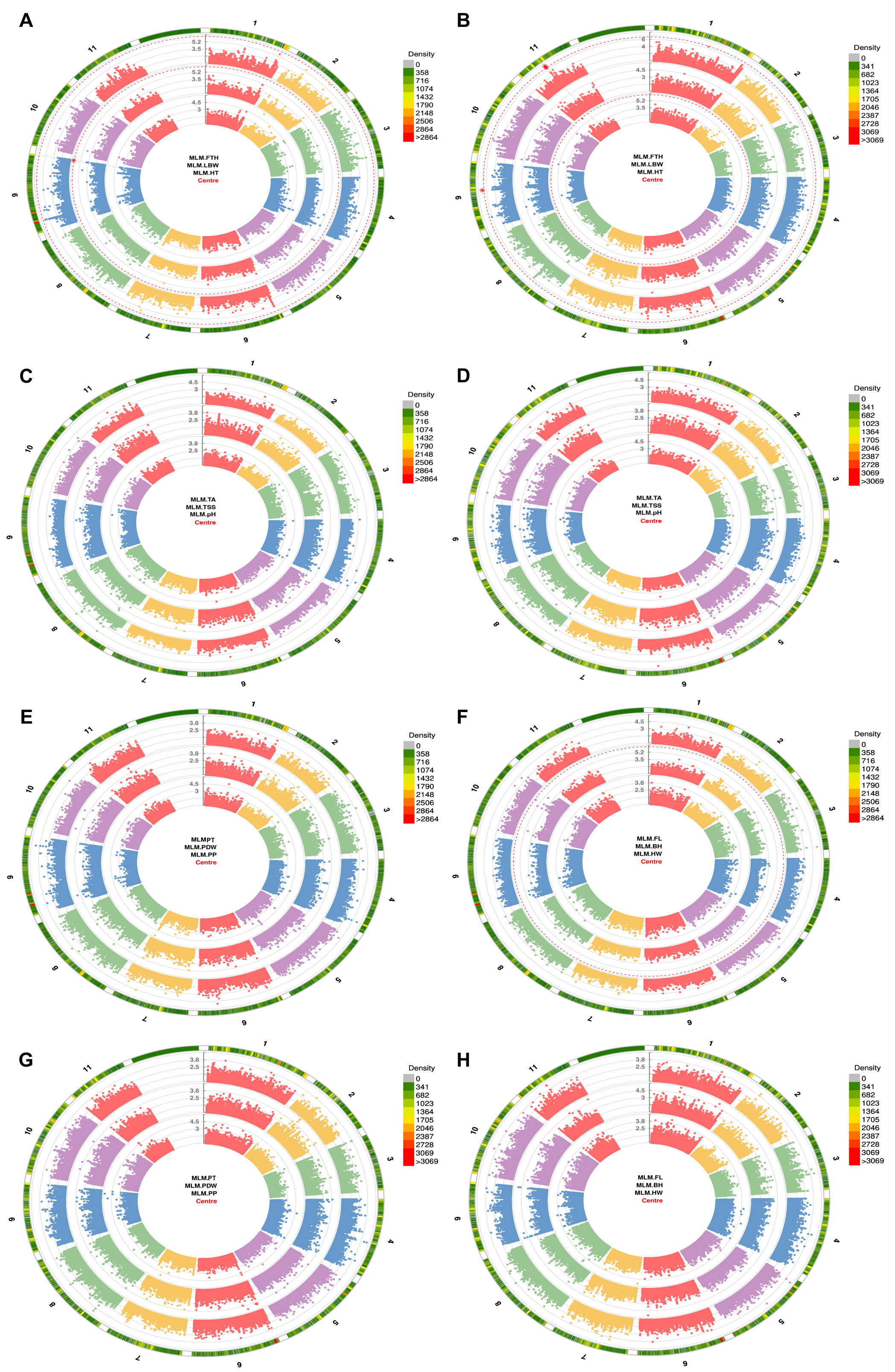

Supplement: jkae108_Supplementary_Data [file jkae108_supplementary_data.zip › Figure_S4_G3-2024-405047.tif]

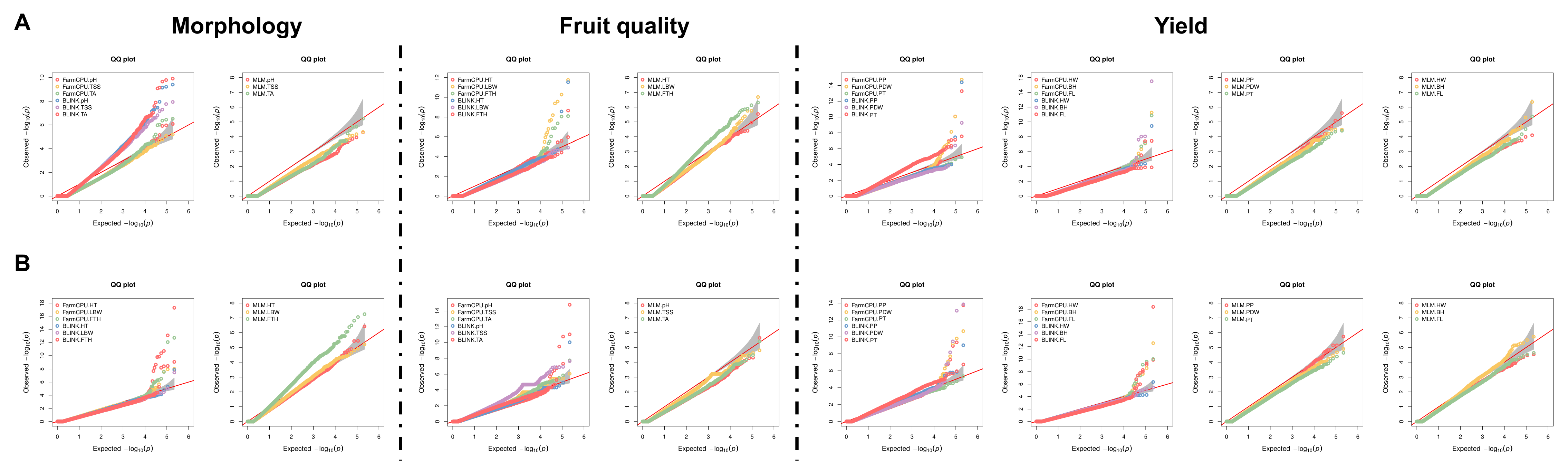

Supplement: jkae108_Supplementary_Data [file jkae108_supplementary_data.zip › Figure_S5_G3-2024-405047.tif]
